# Isolation and characterization of a strain of Lichtheimia corymbifera (ex Absidia corymbifera) from a case of bovine abortion

**DOI:** 10.1186/1477-7827-7-138

**Published:** 2009-11-30

**Authors:** Chiara Piancastelli, Francesca Ghidini, Gaetano Donofrio, Stefano Jottini, Simone Taddei, Sandro Cavirani, Clotilde S Cabassi

**Affiliations:** 1 Dipartimento di Salute Animale, Parma University, via del Taglio 10, 43126 Parma, Italy

## Abstract

**Background:**

Lichtheimia corymbifera (previously Absidia corymbifera) is a filamentous zygomycetes belonging to the order Mucorales and to the family Lichtheimiaceae. Members of genus Lichtheimia spp. are cosmopolitan and ubiquitous in nature. Lichtheimia corymbifera is a recognized agent of diseases in man and animals. In cattle it causes abortion and mastitis. Three cases of bovine abortion occurred in a herd located in the Po Valley. Serological examinations were performed on fetal and mother's blood. One of the aborted fetus was referred to our laboratory. The paper describes the isolation and characterization of Lichtheimia corymbifera from a bovine aborted fetus.

**Methods:**

Serological examinations were performed on fetal and mother's blood. Lesions on fetal tissues and placenta leaded the diagnostic suspect towards a mycotic aetiology. Tissues were then put in culture, and at the same time an histological examination was performed, together with bacteriological and virological tests. The isolate from placenta and fetal tissues was identified and characterized by PCR and RFLP, using the ITS region as a target sequence and AclI restriction site within the amplicon to distinguish Lichtheimia corymbifera among the other fungi.

**Results:**

Serological, bacteriological and virological tests gave aspecific results. Histological examination evidenced numerous PAS positive hyphae within the necrotic cotiledons and numerous fungal nonseptate hyphae to the GMS stain. Colonies with typical morphological features of fungi grew up on Sabouraud agar from fetal skin and placenta. On the developed colonies the microscopic examination has shown a large number of nonseptate hyphae and sporangia consistent with Mucorales. PCR and RFLP allowed the identification of the isolate as Lichtheimia corymbifera.

**Conclusion:**

The present report describes the isolation and the molecular characterisation of a fungal isolate from bovine aborted fetus and placenta. The diagnostic protocol allowed to identify and characterise the strain. This is the first isolation in Italy of Lichtheimia corymbifera in a bovine aborted fetus.

## Background

Bovine abortion is defined as an interruption of pregnancy between the 42^nd ^and 260^th ^day [1]. Among the infectious causes of abortion in cattle are included bacteriological, viral, parasitic and mycotic agents [2]. The knowledge of fungi as abortive pathogens is dated from the last century, and mycotic placentitis is a cause of abortion in cattle worldwide [3].

In the northern hemisphere the incidence of mycotic abortion is sporadic, occurring mainly during the winter periods (November - April), when the cows are usually fed with a large amount of hay [3]. This phenomenon is more evident in conditions of poor aeration and high humidity, which promotes the environmental growth of fungi [4]. The administration of wet stored and mouldy fodder, in which a large amount of fungal spores could be present, is a further risk factor for the mycotic abortion. The fungal spores can penetrate through gastric lesions or the respiratory tract and reach the placenta and the fetus where there are optimal conditions for their full development [5]. The mycotic abortion in cattle usually occurs at the 6^th ^- 8^th ^months of gestation and is often followed by placenta retention. The placenta is thickened and wooden in appearance [6], and haemorrhagic necrotizing placentitis, often associated with thickened yellowish necrotic cotyledons may be present [3,7]. On the fetus skin lesions similar to parakeratotic dermatitis are usually evident, characterized by raised ringworm-like fungal skin plaques and blefaritis [5,8,9].

Different fungi have been isolated from aborted fetuses, belonging to the species *Aspergillus fumigatus *and *Aspergillus nidulans*, *Absidia corymbifera*, *Mortierella wolfii*, *Rhizopus *spp., *Mucor *spp., and *Rhizomucor *spp. [4,10]. Previous reports recorded that > 60% of cases of abortion in the USA were caused by uncomplicated infection with *Aspergillus fumigatus*. Zygomycetes (*Absidia, Mortierella, Rhizomucor, Rhizopus*) accounted for about 20% of cases, and the remaining 20% were caused by a wide range of opportunistic filamentous fungi and yeasts [3]. In Australia *Aspergillus *spp. and *Mortierella wolfii *have been isolated [11], and in New Zealand *Mortierella wolfii *has been considered the major mycotic cause of abortion [12]. In Europe, there are reports of mycotic abortion in Great Britain due to *Aspergillus, Mortierella *and *Absidia *spp. since 1967 [13] and more recently [6,14]. In Italy only cases of mycotic abortion due to *Aspergillus *spp. are reported [15].

In February 2009 three cases of abortion occurred during a single week in a Friesian dairy herd located in the province of Parma (Po Valley), area of production of Parmigiano Reggiano cheese. The herd size was 120 animals (60 lactating), free-stall housing, not vaccinated for infectious abortive agents. The abortions occurred between the 5^th ^and the 6^th ^month of gestation, without apparent symptoms. Signs of illness were not observed in the dams before and after abortions. Of the three aborted fetuses, only one showed skin lesions. It was aborted at the 23^rd ^week of pregnancy and referred to our laboratory for diagnostic investigations. Aim of the work is to describe the isolation and characterisation of a strain of *Lichtheimia corymbifera *isolated from the referred fetus, to provide a complete diagnostic protocol in case of bovine mycotic abortion.

## Methods

### Samples

The aborted fetus and placenta were chilled and referred to our laboratory within 12 hours from the abortion, together with the blood samples from the aborted cows.

### Histological examinations

Histological examinations were performed on fetal tissue samples including skin, heart, lung, liver, spleen, kidney, fetal stomach and placenta collected during the necropsy and fixed in 4% buffered formalin (Bio-Optica). The samples were routinely processed and stained with hematoxylin and eosin (HE) for histopathological evaluation. To investigate the presence of fungi, periodic acid Schiff reaction (PAS) and Grocott's Methenamine Silver stain (GMS) were also performed on tissue sections.

### Culture

Bacteriological examination was performed on fetal spleen, liver, kidney, lung, heart, skin, stomach content and placenta on blood agar (5% bovine erythrocytes) and Mc Conkey agar (DIFCO) and incubated for 24-48 hours at 37°C in air. The fetal stomach content was specifically cultured for the isolation of *Brucella *spp. on 5% blood agar and incubated in microaerophilic (5% CO_2_) environment.

Mycological culture was performed on fetal spleen, liver, kidney, lung, heart and placenta on Sabouraud agar (DIFCO), and incubated for 24-48 hours at 37°C in air.

### Determination of melezitose assimilation

Melezitose assimilation was performed using ID32C strip (bioMérieux, Marcy l'Etoile, France) as reported by Schwarz and coll. [16].

### Serological examinations

Serological examinations for the main infectious abortive agents were performed on blood sera from aborted cows. Moreover, during the necropsy of the fetus, 1 ml of blood from the atrial chambers of the heart was taken. The serological examination of the blood samples to investigate the presence of antibodies against abortive agents was performed for *Brucella abortus *by card test agglutination (Brucelloslide Test, bioMérieux), *Neospora caninum *by indirect immunofluorescence, *Chlamydophila abortus *by ELISA (Civtest Bovis Chlamydia ps., Hipra), Bovine Viral Diarrea (BVD) virus by seroneutralization, *Coxiella burnetii *by ELISA (Chekit Q Fever antibody ELISA test kit, IDEXX), and *Leptospira *serovar *sejroe-hardjo*, *australis-bratislava*, *pomona-pomona*, *icterohaemorrhagiae-copenhageni *by microagglutination test. ELISAs were performed following manufacturer instructions. Seroneutralization, indirect immunofluorescence and microagglutination test were performed following our in-house protocols.

### Virological examination

Virological examination was performed on fetal spleen and lung tissues by direct ELISA (ELISA BVD/MD antigen mix screening, Pourquier) following manufacturer instructions to assess the presence of BVD virus.

### Molecular identification and characterization

Molecular identification and characterization was performed on isolated fungal mycelia. Genomic DNA extraction, PCR and RFLP were performed as described by Machouart and coll. [17]. Specific sense primers for *Rhizopus *spp., *Rhizomucor *spp., *Mucor *spp., and *Lichtheimia corymbifera *(RpL1, 5' TGATCTACGTGACAAATTCT 3'; RmL1, 5' TGATCTACGCGAGCGAACAA 3'; MucL1, 5' TGATCTACGTGACATATTCT 3'; and AbsL1, 5' TGATCTACACGGCATCAAAT 3', respectively) were used with a degenerate antisense primer (MR1, 5' AGTAGTTTGTCTTCGGKCAA 3'). The region selected for the design of primers excluded the amplification of human DNA and other filamentous fungi. Primers Lap (5' GAAACTGCGAATGGCTCATTA 3') and Rap (5' CAATCCAAGAATTTCACCTCT 3') designed to amplify all the fungi and the human DNA were used as positive controls.

The amplicon was also sub-cloned into the 2886 bp pTZ57R/T vector (Fermentas, Glen Burnie, USA) and subjected to RFLP analysis with AclI restriction enzyme.

## Results

### Macroscopic appearance of the fetus and placenta

**Figure 1:**
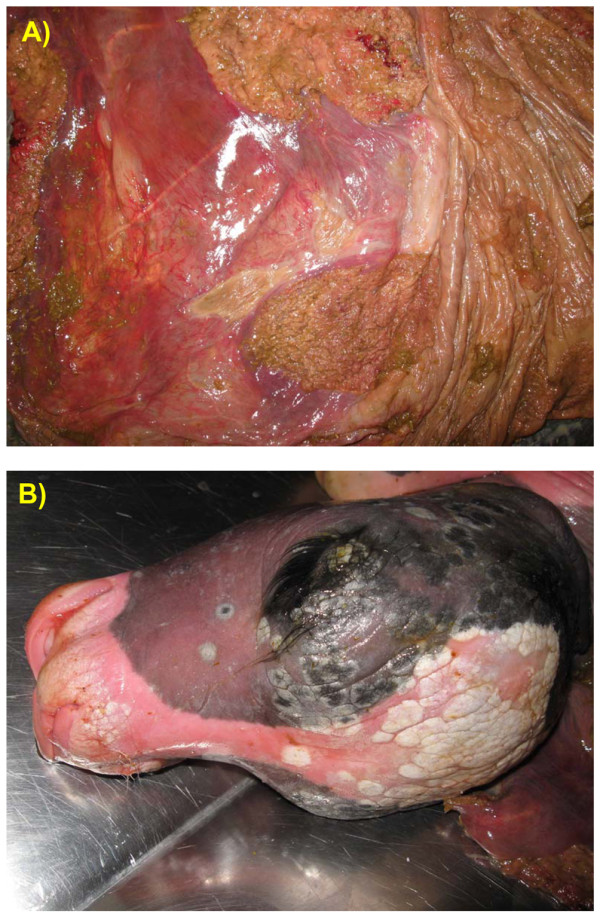
**Gross lesions on the fetus and placenta**. A) The placenta is thickened, cotyledons appear necrotic and firm. Hyperemia of intercotyledonar areas is also visible. B) Aborted calf. White and irregular lesions localized in the head (periorbital region) are visible in the picture.

Cotyledons appeared thickened, firm and necrotic, whereas intercotyledonary chorioallantois showed brown exudate and moderate hyperemia (Fig. 1A). Multifocal and coalescing white, raised and dry plaques were present in the skin of the fetus, mainly localized on the head (periorbital regions) and on the back (Fig. 1B). Small, white, raised plaques were occasionally seen also in the skin of the abdomen, legs and tail. No gross lesions were present in the other organs.

### Finding of fungal hyphae in tissues sections

**Figure 2:**
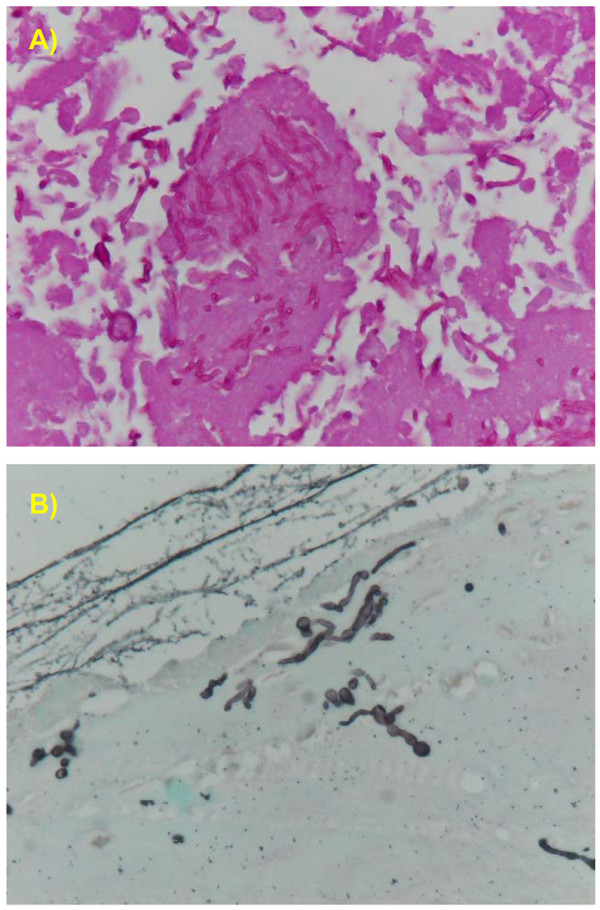
**Histological findings on tissue sections**. Histological section of the placenta. A) Numerous PAS positive hyphae are present within the necrotic cotyledons (PAS reaction, magnification ×200). B) Numerous fungal hyphae (black), irregular in diameter and without regular septation, are present. (GMS stain, magnification ×200).

Microscopically, mild necrotizing placentitis and vasculitis, mainly localized on the cotyledons, were evident in the placenta and in the larger vessels at the base of the cotyledons. Numerous fungal hyphae morphologically consistent with *Zygomycetes *were present in the grossly evident placentar lesions (Fig. 2A and 2B). Hyphae were elongate, filamentous, 6 to 15 μm in diameter and strongly PAS and GMS positive. Skin lesions were characterized by spread multifocal areas of orthokeratotic hyperkeratosis, oedema and rare infiltrate of lymphocytes in the superficial derma.

Rare single hyphae were also present within the skin lesions. No other relevant alterations were detected in the other organs.

### Isolation of fungi

**Figure 3:**
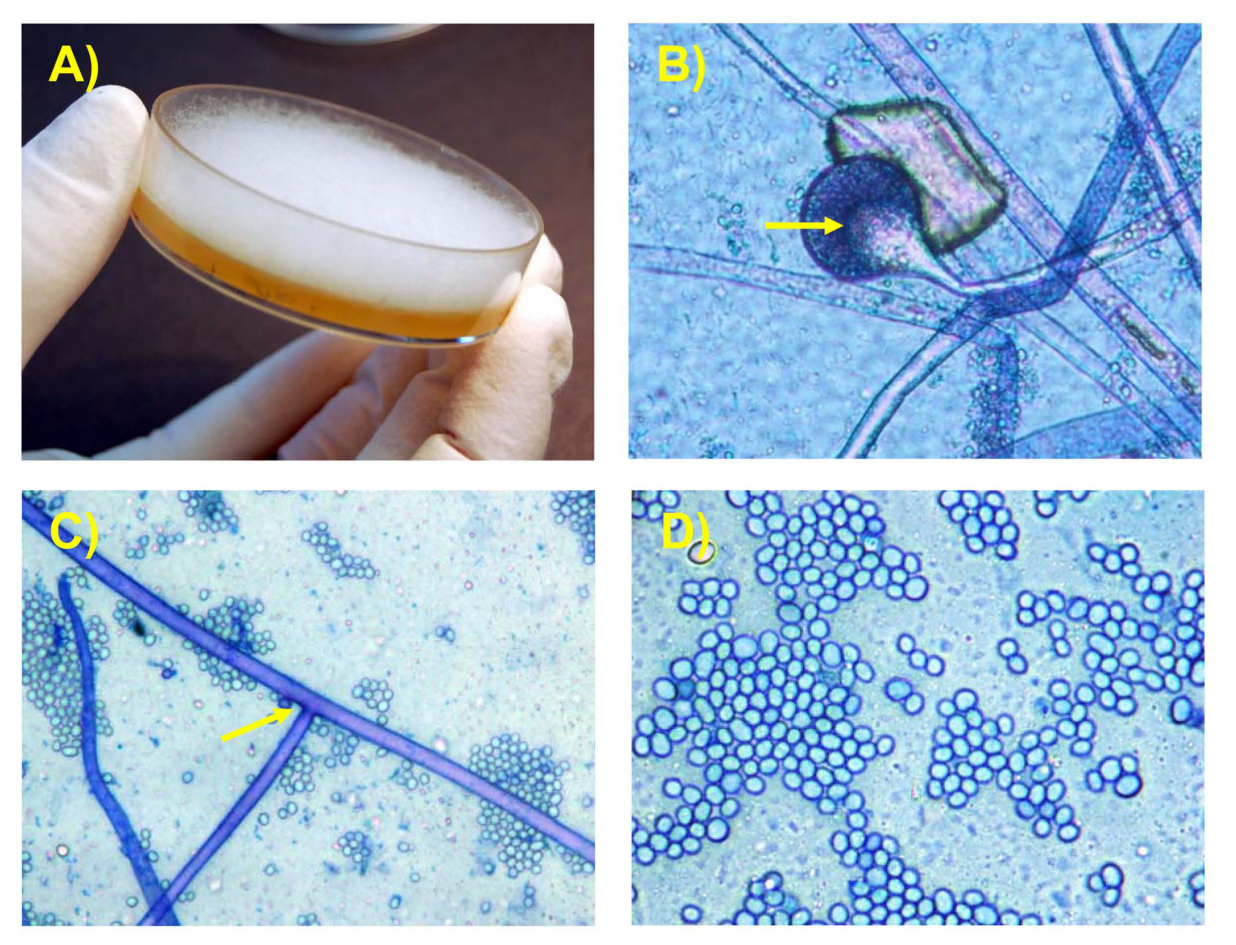
**Cultural and microscopic aspect of the fungus**. A) Fungal culture. The whitish, woolly or cottony aspect of the colonies is evident. The colony raises 1.5 cm high. B) Microscopic aspect of sporangia, pyriform with a conical-shaped columella (indicated by arrow) and pronounced apophysis (Cotton Blue stain, magnification ×400). C) Nonseptate hyphae arising in groups of three at the internode (arrow, magnification ×400). D) Ellipsoidal sporangiospores (magnification ×1000).

Non-haemolytic, non-pathogenic *Escherichia coli *has grown up from liver, spleen, kidney, heart and lung. After 6 days, these plates were still negative for *Brucella *spp. growth. Colonies with typical morphological features of fungi grew up on Sabouraud agar after 24 hours of aerobic incubation from fetal skin and placenta. Colonies were fast growing, white at first becoming pale grey with age, and up to 1.5 cm high (Fig. 3A), woolly to cottony in aspect. The reverse side was uncoloured and without pigment production. On the colonies a direct microscopic examination with Cotton Blue staining showed a large number of nonseptate hyphae, with a diameter of 6 to 15 μm. Specialised hyphae sporangiophores, hyaline or faintly pigmented in colour and simple or branched, arised in groups of three at the internodes (Fig. 3C). Sporangiophores showed relatively small sporangia (20-120 μm of diameter), typically pyriform in shape with characteristic conical-shaped columella and pronounced apophysis (Fig. 3B), often with a short projection at the top, morphologically consistent with *Lichtheimia corymbifera *[18,19]. Sporangiospores were ellipsoid, light grey coloured and smooth-walled (Fig. 3D). They were detected in the sporangium and released to the surrounding area. The presence of rhizoids was not observed. Moreover, the isolate was not capable to utilize melezitose as sole carbon source.

Regarding the serological and virological results, all the blood samples were negative for antibodies against *Leptospira *serovars tested, BoHV-1, *Brucella abortus *and *Chlamydophila abortus*. The mother of the examined fetus was seropositive at low titre for BVDV antibodies. One of the other aborted dams was seropositive for *Neospora caninum *and the third one was seropositive for BVDV, *Neospora caninum *and *Coxiella burnetii*. The serological analyses on the fetal blood sample were negative for the abortive agents tested. The ELISA performed on fetal tissues for BVD virus gave negative result.

### Molecular identification and characterization of the fungal isolate

**Figure 4:**
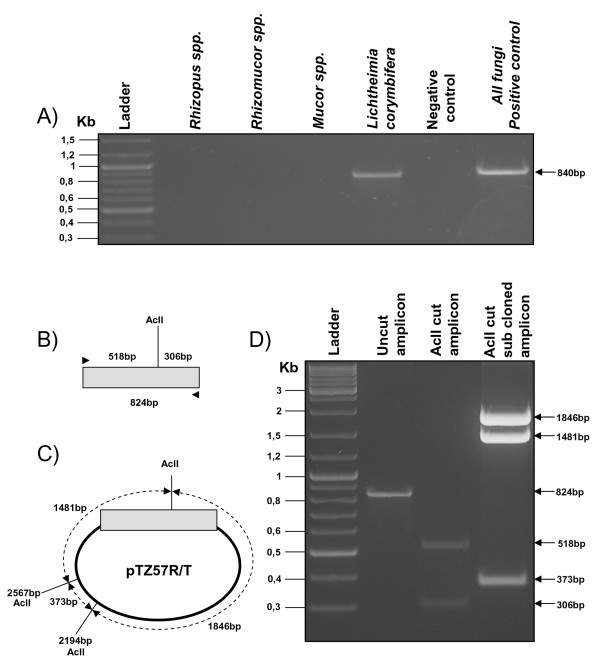
**Molecular characterization of *Lichtheimia corymbifera***. A) Electrophoresis patterns of amplicons obtained with specific sets of primers for *Lichtheimia corymbifera*, *Rhizopus *spp., *Rhizomucor *spp., *Mucor *spp. and a set of primers for all other filamentous fungi used as a positive control (840 bp). B) Diagram showing the amplicon (not on scale) and the AclI restriction enzyme predicted fragments of 518 and 306 bp. C) Diagram showing the sub-cloned 824 bp amplicon (not on scale) into the pTZ57R/T vector and AclI restriction sites within the vector (2194 bp and 2567 bp) and within the amplicon. D) Electrophoresis patterns of AclI uncut 824 bp amplicon, AclI cut 824 bp amplicon and AclI cut sub-cloned 824 bp amplicon.

An expected specific amplicon of 824 bp was obtained only with the set of primers specifying for *Lichtheimia *(ex *Absidia*) *corymbifera*. In contrast none amplification products were obtained when primers for *Rhizopus *spp., *Rhizomucor *spp. and *Mucor *spp. were applied (Fig. 4A). The 824 bp amplicon was sub-cloned into pTZ57R/T vector and both the 824 bp PCR amplicon (Fig. 4B) and the subcloned amplicon (Fig. 4C) were analyzed by RFLP with AclI restriction enzyme. AclI digestion generated 2 restriction fragments of 518 and 306 bp for the 824 bp PCR amplicon and 3 fragments of 1846, 1481 and 373 bp for the subcloned 824 bp amplicon. In both cases the restriction pattern corresponds to *Lichtheimia corymbifera *(Fig. 4D). Sequencing of the 824 bp cloned fragment further confirmed the specificity of the amplicon and the absence of hypothetical polymorphisms or mutations.

## Discussion

In the area where the abortions occurred, the nutrition of dairy cattle is regulated by strict rules and the administration of silage is forbidden. Consequently, dairy cows are fed with a major amount of hay. In our case, heifers were usually fed with grass and hay, while during the pregnancy the ration was supplemented with hay stored the year before in a moist place, exposed to bad weather conditions. The risk factors associated to the administration of mouldy hay were present. Moreover, the macroscopic lesions on the fetus and placenta were consistent with mycotic abortion, confirmed by cultural and histological findings [20]. The morphological aspect of the colonies on Sabouraud agar, the microscopic aspect of the hyphae, sporangia and sporangiospores and the execution of the PCR and RFLP directed the diagnosis to *Lichtheimia corymbifera *[17,18]. The isolation of non-pathogenic *E. coli *from fetal tissues, associated to the absence of gross lesions in the fetal organs, and the BVDV seropositivity at low titre of the dam have to be considered not meaningful to demonstrate the involvement of these pathogens in the abortive event [21]. In addition, necrotizing placentitis is rarely associated to BVDV, and in our case BVD virus was not detected by direct diagnostic procedures on fetal spleen and lungs.

## Conclusion

Our findings support the conclusion that *Lichtheimia corymbifera *is the causative agent of the abortion. The present case is particularly relevant because represents the first isolation in Italy of *Lichtheimia corymbifera *in a bovine aborted fetus. It is useful to stress that in case of abortion the routine diagnostic procedures should include the mycotic component and assess the fungal species [4]. In accordance to the assumption that mycotic abortion is characterised by the presence of mycotic elements associated with placentitis and fetal dermatitis, and that morphologic characteristics of hyphae in tissues have to be compatible with cultured isolates [18,19], confirmation lies in the isolation and identification of the specific mycotic agent. In our case, identification has been confirmed by PCR, and the characterization of the isolate has been made by RFLP. This diagnostic procedure allowed to establish without doubt the etiology of abortion.

## Competing interests

The authors declare that they have no competing interests.

## Authors' contributions

CSC conceived, designed and wrote the paper. CP and FG performed the bacteriological, mycological and serological tests. SJ performed the histological tests. GD was responsible for the molecular identification and characterisation, and intellectually contributed and helped to write the paper. ST performed the serological tests and intellectually contributed and helped to write the paper. SC reviewed the paper. All authors read and approved the final manuscript.
